# Assessment of Community Event–Based Surveillance for Ebola Virus Disease, Sierra Leone, 2015

**DOI:** 10.3201/eid2208.160205

**Published:** 2016-08

**Authors:** Ruwan Ratnayake, Samuel J. Crowe, Joseph Jasperse, Grayson Privette, Erin Stone, Laura Miller, Darren Hertz, Clementine Fu, Matthew J. Maenner, Amara Jambai, Oliver Morgan

**Affiliations:** International Rescue Committee, New York, New York, USA (R. Ratnayake);; Centers for Disease Control and Prevention, Atlanta, Georgia, USA (S.J. Crowe, M.J. Maenner, O. Morgan);; International Rescue Committee, Freetown, Sierra Leone (J. Jasperse, G. Privette, E. Stone, L. Miller, D. Hertz);; Action contre la Faim, Kambia, Sierra Leone (C. Fu);; Sierra Leone Ministry of Health and Sanitation, Freetown (A. Jambai)

**Keywords:** Ebola virus, Ebola virus disease, viruses, surveillance, community event–based surveillance, epidemic, Sierra Leone, community, detection, transmission, epidemiology, West Africa, epidemiologic link, event triggers

## Abstract

In 2015, community event–based surveillance (CEBS) was implemented in Sierra Leone to assist with the detection of Ebola virus disease (EVD) cases. We assessed the sensitivity of CEBS for finding EVD cases during a 7-month period, and in a 6-week subanalysis, we assessed the timeliness of reporting cases with no known epidemiologic links at time of detection. Of the 12,126 CEBS reports, 287 (2%) met the suspected case definition, and 16 were confirmed positive. CEBS detected 30% (16/53) of the EVD cases identified during the study period. During the subanalysis, CEBS staff identified 4 of 6 cases with no epidemiologic links. These CEBS-detected cases were identified more rapidly than those detected by the national surveillance system; however, too few cases were detected to determine system timeliness. Although CEBS detected EVD cases, it largely generated false alerts. Future versions of community-based surveillance could improve case detection through increased staff training and community engagement.

## Abstract 2

Case detection improved, but many false alerts were generated, suggesting a need for additional staff training.

Community event–based surveillance (CEBS) systems have been used for case finding during outbreaks and to increase sensitivity for detection of diseases targeted for eradication (*1**–**5*), but this type of surveillance has not been implemented rapidly on a national scale during a large health emergency. In October 2014, near the peak of the Ebola virus disease (EVD) epidemic in West Africa, the International Rescue Committee, Sierra Leone’s Bo District Health Management Team, and the US Centers for Disease Control and Prevention developed CEBS in Sierra Leone to serve as a village-level active surveillance system for reporting possible EVD cases (*6*). At that time, many infected persons were not detected until after they had died by the national surveillance system, which consisted of contact tracing, healthcare facility surveillance, and a telephone hotline for reporting events; thus, opportunities for virus transmission were prolonged (*6*). CEBS was designed to supplement the national surveillance system by training community members to identify, within their own communities, unsafe burials and persons with signs and symptoms compatible with EVD infection. Through its community presence, CEBS was positioned to detect EVD cases that were not epidemiologically linked to other confirmed cases at the time of detection; identification of such cases could provide early warning of new chains of transmission.

A brief pilot study in Bo District during November and December 2014 demonstrated that community leadership accepted CEBS and that CEBS could identify possible EVD cases. Thus, the Ebola Response Consortium, led by the International Rescue Committee and consisting of 15 humanitarian organizations committed to stopping the Ebola virus epidemic, worked with the Sierra Leone Ministry of Health and Sanitation to implement CEBS in 9 of Sierra Leone’s 14 districts (Figure 1). CEBS began operations on February 27, 2015, when the surveillance system recorded its first alert.

**Figure 1:**
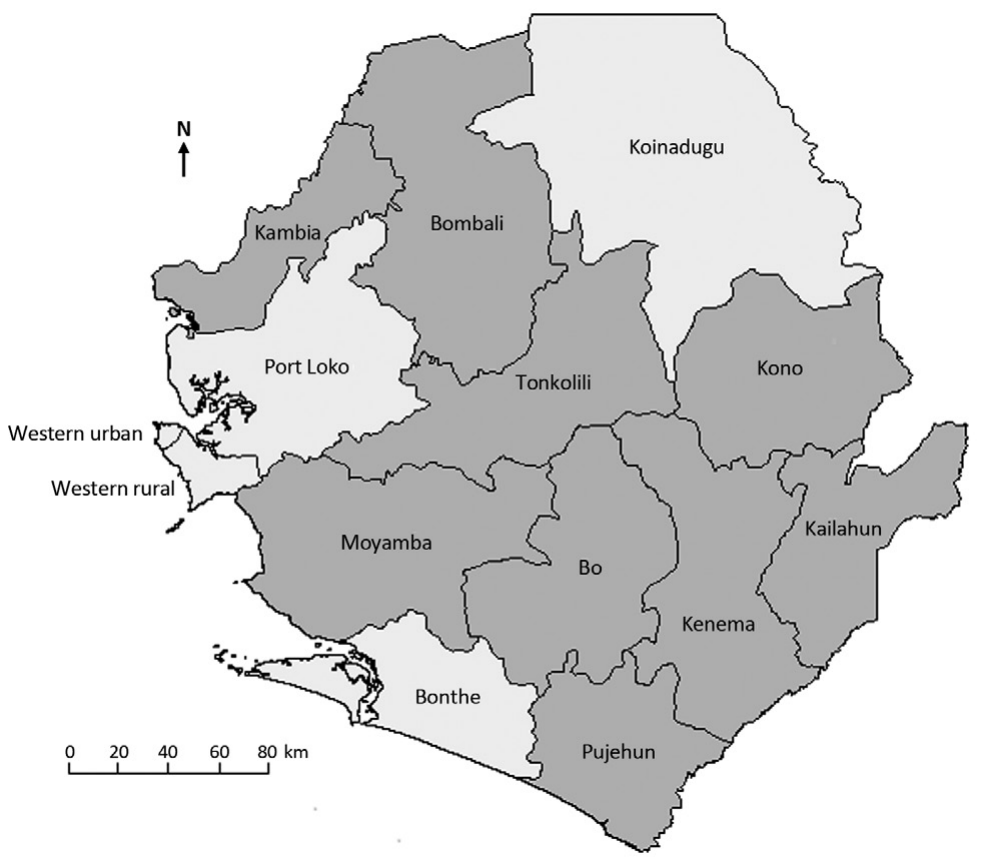
Nine districts (dark gray shading) where community event–based surveillance for Ebola virus disease was operational, Sierra Leone, February 27–September 30, 2015.

During February 27–September 30, 2015, we evaluated the ability of CEBS to detect possible EVD cases and unsafe burials in the 9 districts. We also conducted a subanalysis of the system during its first 6 weeks of operation in Kambia District to assess the sensitivity, positive predictive value (PPV), and timeliness of case detection for persons with no epidemiologic links at the time of detection. Among the districts in which CEBS was operational, Kambia District was the only one that experienced ongoing active virus transmission during the subanalysis period.

## Methods

### Data Collection, Data Flow, and Reporting

The CEBS system included community health monitors, community surveillance supervisors, and community health officers, each of whom received job-specific training in the month before beginning operations. Formal evaluations of staff knowledge were not conducted due to the rapid nature of the system deployment, but some districts provided informal refresher training when possible. Community health monitors were volunteers or existing community health workers who were trained to detect 6 trigger events suggestive of Ebola virus transmission: 1) >2 sick or dead members in a household, 2) a sick or dead person after an unsafe burial or corpse washing, 3) a sick or dead health worker or traditional healer, 4) a sick or dead traveler, 5) a sick or dead contact of an EVD patient, or 6) an unsafe burial or corpse washing. A seventh category, “other,” was included so that community health monitors could report and describe other unusual events that did not fall under any of the 6 defined trigger events. Community health monitors reported events to their community surveillance supervisors via mobile telephone calls; the supervisors then conducted preliminary investigations. The community health officers, who were trained professionals within the national public health system, often assisted surveillance supervisors with preliminary investigations, but some delegated that responsibility to the supervisors and only assisted when needed. The surveillance supervisors or community health officers then reported events that remained suspect to their local District Ebola Response Center for follow-up.

Community health monitors were responsible for their own village and sometimes, to help ensure adequate coverage, a few small villages within walking distance of where they lived. Surveillance supervisors were assigned a larger area but were provided with motorcycles to facilitate investigations. All CEBS staff were given a mobile telephone with monthly phone credit or a subscription in a prepaid, closed user-group network. Community health monitors were expected to immediately contact their surveillance supervisors to report alerts and to contact them weekly to confirm the absence of reportable events.

When a surveillance supervisor received an alert from a community health monitor, the supervisor recorded the alert information on a standardized form, a weekly alert log. The following data were captured: date; time; trigger event involved; type of alert (sickness, death, unsafe burial, or other); and age, sex, and location of the person(s) concerned. The surveillance supervisor also recorded what, if any, actions were taken to respond to the alert; whether the alert was raised to the District Ebola Response Center; and whether local social mobilization teams were notified to provide health education activities.

Surveillance supervisors submitted alert logs to the CEBS district lead at the end of each week. On a weekly basis, the district lead entered the data into a standardized spreadsheet, checked for duplicate reporting, and submitted the document to the CEBS coordination unit in Freetown, Sierra Leone. The district lead also cross-checked each CEBS alert against those in the District Ebola Response Center alert records and confirmed the final alert status as 1) the identified illness or death did not meet the suspected case definition, 2) the alert involved a suspected or probable case-patient who tested negative, or 3) the alert identified a confirmed case. In Sierra Leone, a suspected case-patient was defined as 1) a person with a fever (temperature >38°C) who was a known contact of a suspected, probable, or confirmed EVD clinical case-patient; 2) a person with >3 EVD-compatible symptoms (e.g., headache, vomiting, and diarrhea) and who had had contact with a clinical case-patient; 3) a person with fever and >3 EVD-compatible symptoms; 4) a person with inexplicable bleeding or miscarriage; or 5) a deceased person with an unexplained death. A probable EVD case-patient was defined as a person who was determined likely to have EVD based on clinical or epidemiologic factors. A confirmed case-patient was defined as a person who tested positive for Ebola virus RNA by quantitative reverse transcription PCR or a similar diagnostic test (*7*).

By September 30, 2015, CEBS had been implemented by 7,416 community health monitors and 137 surveillance supervisors across 9 districts in Sierra Leone (Figure 1). Implementation was undertaken by the International Rescue Committee (in Bo, Kenema, Kono, and Tonkolili Districts); Save the Children International (in Kailahun and Pujehun Districts); CARE International (in Bombali District); Action contre la Faim (in Kambia and Moyamba Districts); and ABC Development (in Kambia District). The Ebola Response Consortium provided technical assistance for system implementation and operations.

As a new surveillance system, CEBS had no trained staff, field equipment (e.g., telephones and motorbikes), or reporting infrastructure. The startup costs were estimated at US$1.3 million. Once the system was operational, the monthly costs were ≈US$129,000, which covered training, telephones, motorbikes, fuel, and incentives.

### Methods of Evaluation

We described the alerts by type (illness, death, unsafe burial, and other) and by trigger event used. We calculated alert rates and death rates per 100,000 persons per day by district, using 2004 district population estimates (*8*). The sensitivity of CEBS for case detection was assessed using the Ministry of Health and Sanitation’s surveillance data (*9*). Sensitivity was evaluated by dividing the number of CEBS-detected confirmed cases by the total number of confirmed cases detected by the overall surveillance system. PPV of confirmed case detection was determined by dividing the confirmed cases detected by CEBS by the suspected, probable, and confirmed cases detected by CEBS.

During April 13–May 30, 2015 (i.e., from the date CEBS first became operational in Kambia to the end date of our team’s field investigations), we evaluated cases in persons in Kambia District who had no identified epidemiologic links at the time of detection. We used the Ministry of Health and Sanitation’s Epi Info database to identify all confirmed cases in Kambia during the evaluation period (*10*). The database served as the line list of suspected, probable, and confirmed cases in Sierra Leone and integrated information from the epidemiologic investigation, including date of symptom onset and potential risk factors. By interviewing frontline public health and CEBS staff, we were able to determine whether and how CEBS was involved in case detection. We considered case-patients to have no identified epidemiologic links at the time of detection if they were not on the contact list used by the district contact tracers and, therefore, were not being actively monitored. The timeliness of detection of these cases was determined by calculating the interval in days between the date of symptom onset and the date of detection.

This assessment was a part of a nonresearch public health response activity and thus did not undergo institutional review board review. In addition, we used only information that had already been collected for public health surveillance purposes, so informed consent was not obtained.

## Results

**Figure 2:**
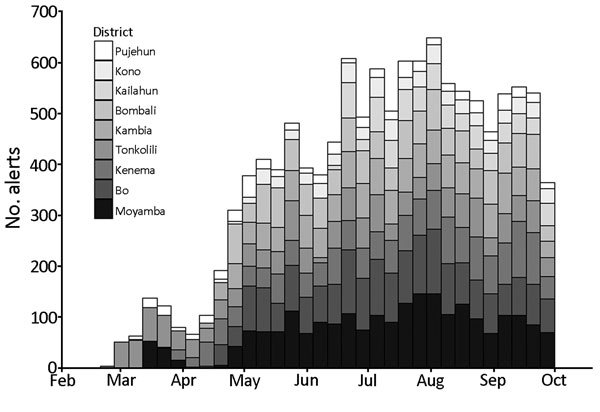
Weekly alerts from community event–based surveillance for Ebola virus disease, by district, Sierra Leone, February 27–September 30, 2015.

During February 27–September 30, 2015, a total of 12,126 alerts were reported through CEBS in 9 Sierra Leone districts (Figure 2). Tonkolili was the first district to report on a consistent basis, beginning on February 27, followed by Moyamba (March 6), Pujehun (March 14), Kenema (March 31), Kambia (April 13), Bo (April 18), Kono (April 23), Bombali (April 27), and Kailahun (June 14). From June 14 onward, the districts were collectively reporting an average of 79 alerts per day.

Of the 12,126 alerts reported, 86% (10,421) were for deaths, 14% (1,646) for illnesses, and <1% for unsafe burials (7) or other (52) (Table 1). The alert rate per 100,000 persons per day differed by district and alert type. Of note, Moyamba and Kambia generated the highest rates of death alerts (3.81 and 2.19, respectively), and Kailahun and Bombali reported the highest rates of sick alerts (0.67 and 0.32, respectively) (Table 2). The CEBS death reporting rates were substantially lower than the expected crude death rate of 4.66 deaths/100,000 persons/day used by the World Health Organization (*11*).

**Table 1:** CEBS for Ebola virus disease, Sierra Leone, 2015*

Surveillance variable	No. (%)
Alerts, n = 12,126	
Death	10,421 (86)
Sickness	1,646 (14)
Unsafe burial	7 (<1)
Other†	52 (<1)

Trigger events	
>2 persons sick or dead in household	205 (2)
Sickness or death after unsafe burial	59 (<1)
Sickness or death in HCW	70 (<1)
Sickness or death in traveler	191 (2)
Sickness or death in contact of case-patient	36 (<1)
Unsafe burial or washing of corpse	7 (<1)
Other‡	11,558 (95)

Cases	
Suspected, probable, or confirmed	287 (2)
Tested and ruled negative	271
Confirmed	16
Did not meet case definition	10,173 (84)
Not escalated as an alert	774 (6)
Lost to follow-up§	892 (7)

*This analysis was conducted during February 27–September 30, 2015. CEBS, community event–based surveillance; HCW, healthcare worker. †Alerts for unusual events that did not fall under any of the first 6 events listed in the trigger events section of the table. ‡A total of 10,042 (86.9%) of these events were deaths in the community. §No follow-up or missing information on follow-up.

**Table 2:** CEBS alert rates by type and district, Sierra Leone, February 27–September 30, 2015*

District	Population estimate†	Days of CEBS operation	Death alerts			Sick alerts	
			Total no. alerts	Rate‡		Total no. alerts	Rate‡
Moyamba	278,119	208	2,203	3.81		74	0.13
Bombali	494,139	156	1,137	1.47		250	0.32
Kambia	341,690	170	1,273	2.19		148	0.25
Bo	654,142	165	1,775	1.64		238	0.22
Tonkolili	434,937	215	1,343	1.44		192	0.21
Kenema	653,013	183	1,327	1.11		308	0.26
Kono	325,003	160	573	1.10		48	0.09
Pujehun	335,574	200	432	0.64		108	0.16

Kailahun

465,048

108

358

0.71

339

0.67

*CEBS (community event–based surveillance) was conducted in 9 of the country’s 14 districts. †Estimates from the 2004 Population and Housing Census: Analytical Report on Population Projection for Sierra Leone (*8*). ‡No. alerts/100,000 persons/d.

In terms of the 6 defined trigger events, the most commonly cited was >2 sick or dead household members (n = 205, 2%). Sickness or death of a traveler was the second most cited (n = 191, 2%). In total, the 6 defined trigger events accounted for <5% of the alerts (Table 1). The seventh trigger event category (i.e., other) accounted for the most alerts (n = 11,558, 95%); a total of 10,042 (87%) of the alerts categorized as other were for deaths in the community (Table 1). Surveillance supervisors and community health officers escalated 93% of the alerts to the District Ebola Response Centers for follow-up.

A total of 287 (2%) of all persons who triggered alerts met the suspected, probable, or confirmed case definitions. Of these 287 persons, 215 (75%) were detected after death, and 271 (94%) tested negative for EVD. During the study period, 16 confirmed EVD cases were reported by CEBS in Kambia (n = 13) and Tonkolili (n = 3); half of the infected persons were detected while alive. During this same period, the Ministry of Health and Sanitation documented 53 confirmed cases in the CEBS districts. Overall, the sensitivity for confirmed case detection by CEBS was 30% (16/53 confirmed cases), and the PPV was 6% (16/287 suspected, probable, or confirmed cases). Sensitivity was 27% (13/49 confirmed cases) in Kambia and 75% (3/4 confirmed cases) in Tonkolili; PPV was 7% (13/175 suspected, probable, or confirmed cases) in Kambia and 9% (3/33 suspected, probable, or confirmed cases) in Tonkolili.

During the 6-week subanalysis in Kambia, the Ministry of Health and Sanitation database identified 13 confirmed EVD cases in the district. CEBS staff reported 8 of these patients, of whom 7 were alive at the time of the alert. Upon further investigation, we found that 3 of the cases were reported by community health monitors who also served as contact tracers through the contact tracing reporting system. Therefore, CEBS was not the main reporting source for these 3 cases, and the cases were not recorded in the CEBS database or counted as cases detected by CEBS. For the remaining 5 cases, 3 were classified as other trigger events, 1 was in a sick contact of a confirmed case-patient, and 1 was a sick member of a household.

Six of the 13 confirmed case-patients in Kambia had no epidemiologic links when they were detected; CEBS staff identified 4 of these case-patients. The time from symptom onset to detection ranged from 1 to 3 days for the 4 cases identified by CEBS and was 5 and 7 days, respectively, for the 2 cases detected by other components of the national surveillance system (Table 3). For the latter 2 cases, 1 of the ill persons lived in a village that was not covered by a community health monitor at the time of detection, and the case was identified after the person had died; the other person resided in a CEBS-covered village, but the case was not detected until the ill person was admitted to a local hospital.

**Table 3:** Timeliness of identification of confirmed Ebola virus disease cases with no known epidemiologic links to other confirmed cases at detection, Kambia District, Sierra Leone, 2015*

Detected by CEBS	Patient age, y/sex	Symptom onset date	Detection date	Days from onset to detection
Yes	52/M	Apr 17	Apr 20	3
No	45/M	Apr 17	Apr 22	5
Yes	23/F	Apr 23	Apr 25	2
Yes	25/F	Apr 24	Apr 27	3
No	56/F	Apr 23	Apr 30	7
Yes	29/M	May 28	May 29	1

*The data are for April 13–May 30, 2015. CEBS, community event–based surveillance.

During the subanalysis in Kambia, surveillance staff from other districts indicated that the CEBS network had detected additional outbreaks that were not caused by Ebola. Community health monitors identified 2 measles clusters in Kono and 1 measles cluster in Bombali, leading to the initiation of investigations and implementation of control measures, including isolation of ill persons and vaccination of susceptible children. Community health monitors in Bombali and Kono also reported suspected chickenpox clusters.

## Discussion

Our evaluation indicates that, during its period of operation, CEBS effectively generated alerts for and detected nearly one third of all EVD cases found in its districts. Although this would rightly be considered a low sensitivity for an independent surveillance system, CEBS was designed to supplement a larger, established system. The low PPV of CEBS also was expected because of the tendency of event-based systems to provide higher sensitivity while generating a large number of false alerts (*12*) and because there were few true EVD cases. Ruling out EVD in times of low transmission requires investigation of all alerts and isolation and testing of all suspected case-patients (*13*), most of whom will be determined to be uninfected.

Our data from the subanalysis in Kambia are too few to draw a meaningful conclusion, but they suggest that CEBS may be capable of quickly finding cases with no identified epidemiologic links. If this is true, the system could be used to detect the early stages of new infectious disease outbreaks or to rapidly identify the spread of disease to new geographic areas during ongoing outbreaks or epidemics. Nevertheless, even within this small sample of cases, CEBS failed to detect 2 cases with no known epidemiologic links, which highlights the need for adequate coverage of villages by community health monitors, development of stronger links between communities and health monitors, and vigilance by the monitors.

One unexpected finding was that CEBS detected a large number of deaths in the community. Although not intended to serve as a reporting system for community deaths, CEBS did contribute to death reporting, which was a major initiative of the national government and social mobilization programs. By detecting dead bodies that were then tested and found to be negative for Ebola infection, CEBS helped to confirm the lack of virus transmission, thereby providing some evidence that the epidemic had, in fact, ended in a given district. However, death reports are a late indicator of infection and, thus, do not enable isolation of patients early in the disease course, a control measure that could result in reduced transmission (*14**–**16*). The death reporting rates also were considerably lower in most CEBS districts than would be expected based on estimated death rates (*11*); consequently, CEBS reporting rates were not a substitute for death surveillance or registration.

Another unexpected finding was the detection of 3 measles outbreaks. Given extensive undervaccination and undertreatment of other communicable diseases during the epidemic, it was expected that a large number of disease outbreaks would go undetected (*17**,**18*), but we did not anticipate that CEBS would detect a few of them. CEBS staff might have detected these clusters because some of the trigger events, such as >2 sick or dead persons in a household, were not specific to EVD. The staff also might have identified the clusters because they were looking for signs of illness in their communities, irrespective of the cause or signs or symptoms. This finding provides some indication that community-based surveillance could be used to provide early warning of a variety of diseases of public health concern.

Our evaluation also revealed several critical weaknesses in CEBS, some of which may be due to the rapid implementation of the system. First, community health monitors primarily relied on the trigger category other to classify community deaths and alerts, rather than the defined trigger events that they were taught to seek out. This lack of use of defined trigger events could imply that some of the triggers were not sensitive enough to capture Ebola virus transmission. It is also possible that the staff miscategorized the alerts and that many alerts did, in fact, fit a trigger event category. However, most alerts categorized as other were not reported with sufficient information to assess whether they fit a defined trigger event category. Before future systems are widely implemented, the validity of triggers should be more rigorously tested, and refresher training of staff should be regularly provided to reinforce trigger event recall. Rapid implementation at scale is difficult to achieve while also providing comprehensive training and developing strong links between the community and the surveillance team. Ideally, community-based surveillance should be developed and implemented when a large outbreak is not underway. Such a system would then be in place and available for adaptation if a public health crisis arises.

Another weakness of CEBS is that community health monitors reported relatively few illnesses, which is concerning for a system that aimed to detect illness quickly to reduce opportunities for virus transmission. The low proportion of illness alerts may indicate that the intended meaning of illness was unclear or, more likely, that community health monitors were concerned about negative consequences from the community for reporting an event, particularly if the affected person was not infected with Ebola virus.

A final weakness of note is that CEBS detected few unsafe burials. This lack of reporting could reflect the general challenge faced by EVD surveillance in exposing a cultural tradition that communities intentionally guard closely. However, by February 2015, when awareness of the Ebola virus transmission risks of traditional burials were more fully understood, community members may have bypassed their community health monitors to relay information about unsafe burials directly to the preexisting burial management alert system (*19*).

Our evaluation has several limitations. First, we conducted the evaluation during a period of low Ebola virus transmission; therefore, we cannot draw conclusions about how CEBS would perform in a high-transmission environment. Second, the subanalysis in Kambia lasted only 6 weeks and involved only a few cases. Consequently, the results regarding the ability of CEBS to find cases with no identified epidemiologic links at the time of detection cannot be considered conclusive. Additional implementation and evaluation of CEBS in future EVD outbreaks would provide data to assess the relative merits of this approach. Third, we could not analyze the sensitivity of CEBS to detect other disease outbreaks because no reference standard exists to inform the denominator, and no reporting mechanism exists within CEBS to inform the numerator. Fourth, CEBS was implemented primarily in rural settings, so we do not know how the system would perform in a densely populated urban setting, such as a capital city. Last, an anthropologic understanding of the lack of illness and burial reporting would inform a more comprehensive interpretation of these results.

The Sierra Leone Ministry of Health and Sanitation plans to use community-based surveillance as part of its Integrated Disease Surveillance and Response system, which is tasked with detecting and responding to several priority diseases, conditions, and events (*20*). Community-based surveillance could extend disease surveillance beyond district health clinics to the village level and provide an early warning function. This would contribute to meeting the core capacity requirements of the International Health Regulations to detect and report disease at the community level to facilitate the immediate implementation of control measures before an outbreak expands further (*21**,**22*). Nonetheless, our evaluation reveals several challenges that should be addressed. Detailed assessments should be undertaken to determine how community health monitors recognize and categorize symptomatic illness and the barriers to their ability and willingness to report illness. The assessment results should then be applied to refine trigger definitions and processes. Given that valid disease measures are the basis of an effective surveillance system, these issues are the most pressing ones that need to be studied and addressed to strengthen future iterations of community-based surveillance. In addition, trigger definitions should remain simple to ensure that community health monitors can understand and correctly apply them, which may mean that a few salient, event-based triggers would be more effective than several case-based, specific triggers. Alternatively, more extensive, regularly repeated training of community health monitors might be needed to ensure adequate recall and reporting of more complicated triggers. Furthermore, to sustain efficacy and performance, community-based surveillance must be fully integrated into the overall surveillance system and adequately supported to ensure response capacity.
